# Spatio-temporal selection of reference genes in the two congeneric species of *Glycyrrhiza*

**DOI:** 10.1038/s41598-020-79298-8

**Published:** 2021-03-02

**Authors:** Yuping Li, Xiaoju Liang, Xuguo Zhou, Yu An, Ming Li, Ling Yuan, Yongqing Li, Ying Wang

**Affiliations:** 1 Key Laboratory of South China Agricultural Plant Molecular Analysis and Genetic Improvement and Guangdong Provincial Key Laboratory of Applied Botany, South China Botanical Garden, Chinese Academy of Sciences, Guangzhou, 510650 China; 2 Center of Economic Botany, Core Botanical Gardens, Chinese Academy of Sciences, Guangzhou, 510650 China; 3 University of Chinese Academy of Sciences, Beijing, 100049 China; 4 Department of Entomology, University of Kentucky, Lexington, KY 40546 USA; 5 Institute of Desertification Control, Ningxia Academy of Agriculture and Forestry Sciences, Yinchuan, 750002 China; 6 Department of Plant and Soil Sciences, University of Kentucky, Lexington, KY 40546 USA; 7 Gannan Normal University, Ganzhou, 341000 Jiangxi People’s Republic of China

**Keywords:** Plant development, Secondary metabolism

## Abstract

*Glycyrrhiza*, a genus of perennial medicinal herbs, has been traditionally used to treat human diseases, including respiratory disorders. Functional analysis of genes involved in the synthesis, accumulation, and degradation of bioactive compounds in these medicinal plants requires accurate measurement of their expression profiles. Reverse transcription quantitative real-time PCR (RT-qPCR) is a primary tool, which requires stably expressed reference genes to serve as the internal references to normalize the target gene expression. In this study, the stability of 14 candidate reference genes from the two congeneric species *G. uralensis* and *G. inflata*, including *ACT*, *CAC*, *CYP*, *DNAJ*, *DREB*, *EF1*, *RAN*, *TIF1*, *TUB*, *UBC2*, *ABCC2*, *COPS3*, *CS*, *R3HDM2*, were evaluated across different tissues and throughout various developmental stages. More importantly, we investigated the impact of interactions between tissue and developmental stage on the performance of candidate reference genes. Four algorithms, including geNorm, NormFinder, BestKeeper, and Delta Ct, were used to analyze the expression stability and RefFinder, a comprehensive software, provided the final recommendation. Based on previous research and our preliminary data, we hypothesized that internal references for spatio-temporal gene expression are different from the reference genes suited for individual factors. In *G. uralensis*, the top three most stable reference genes across different tissues were *R3HDM2*, *CAC* and *TUB*, while *CAC*, *CYP* and *ABCC2* were most suited for different developmental stages. *CAC* is the only candidate recommended for both biotic factors, which is reflected in the stability ranking for the spatio (tissue)-temporal (developmental stage) interactions (*CAC*, *R3HDM2* and *DNAJ*). Similarly, in *G. inflata*, *COPS3*, *R3HDM2* and *DREB* were selected for tissues, while *RAN*, *COPS3* and *CS* were recommended for developmental stages. For the tissue-developmental stage interactions, *COPS3*, *DREB* and *ABCC2* were the most suited reference genes. In both species, only one of the top three candidates was shared between the individual factors and their interactions, specifically, *CAC* in *G. uralensis* and *COPS3* in *G. inflata*, which supports our overarching hypothesis. In summary, spatio-temporal selection of reference genes not only lays the foundation for functional genomics research in *Glycyrrhiza*, but also facilitates these traditional medicinal herbs to reach/maximize their pharmaceutical potential.

## Introduction

Licorice or liquorice is the common name of *Glycyrriza uralensis* Fischer, *Glycyrrhiza glabra* Linné or *Glycyrrhiza inflata* Batalin, which are herbaceous perennial plants of the bean family Fabaceae native to the western Asia and southern Europe^1^. Besides its ecological values for windbreak and sand fixation, both roots and shoots of licorice compose specialized bioactive compounds/molecules with pharmaceutical potential. Licorice root extracts have been used in herbalism and traditional medicine and presented anti-carcinogenic^2,3^, anti-inflammatory, anti-fungal, anti-piroplasmic and cytotoxic activities^4^. More recently, glycyrrhizin, the most important bioactive triterpenoid saponin in licorice roots, is under the consideration for treating COVID-19 infection caused respiratory syndrome^5^. And licorice shoots are a kind of high-quality forage grass because of their high content of coarse fiber and flavonoid^6^. Their beneficial effects on human health has made licorice a valuable trade item. However, these bioactive compounds in licorice with pharmaceutical interest, such as glycyrrhizin or flavonoid, are typically in minute quantities^7^. Therefore, a better understanding of pathways associated with biosynthesis, regulation, and accumulation of these phytochemicals becomes a key step to reach the pharmaceutical potential of licorice^8–10^.

Spatio-temporal gene expression is the activation of genes within specific tissues of an organism at specific times during development (WIKIPEDIA). Many key genes only express in certain tissues and at certain developmental stages in response to both internal and external cues to ensure the accomplishment of each step in plant life cycles^11,12^. In addition, accumulation of valuable bioactive constituents in many functional plants is tissue-specific and meanwhile, only happens at specific developmental stages. One well-known example are ginsenosides in ginseng, which accumulate specifically in roots and rhizomes in “mature” plants, while little could be detected at juvenile stage in these perennial plants^13^. Consistently, the expression of regulatory genes and biosynthetic genes of ginsenosides is also spatio-temporal specific^14^. A growing number of studies have been conducted to screen genes involved in the same metabolic pathways by co-expression network^15,16^. Spatio-temporal gene expression profiling may provide important clues for future functional analyses of genes in non-model organisms.

Reverse transcription quantitative real-time PCR (RT-qPCR) is a method for accurate expression analysis and comparisons of small numbers of genes among various experimental samples^17,18^. Because of the accuracy and sensitivity of RT-qPCR suitable internal references for data normalization in RT-qPCR analysis are prerequisite to obtain reliable results^19^. Expression of suitable reference genes should be constant under the experimental conditions to be tested in a specific research^19^. Housekeeping genes, due to their stability and indispensable function for survival, are the typical first choice for reference gene selection^20,21^, and stably expressed genes in RNA-seq experiments might also be good candidates. That being said, no “universal” reference gene has been verified to be stably expressed across all given experimental conditions^22,23^. Consequently, selection of appropriate reference genes is required for a standardized RT-qPCR procedure following the MIQE (Minimum Information for publication of Quantitative real time PCR Experiments) guidelines^24^.

In licorice, the types and contents of many bioactive compounds varied remarkably among different tissues at different developmental stages. Glycyrrhizin, the most important bioactive triterpenoid saponin in licorice roots, are predominantly accumulated in roots and rhizomes^25^, and accumulated to higher levels in summer than in winter^26^. Spatio-temporal spcific expression of related biosynthetic and regulatory genes should be the cause of these spatial-temporol accumulation of bioactive compounds. For example, the key genes in glycyrrhizin biosynthetic pathway, *β*-amyrin synthase (*β*-AS), *β*-amyrin 11-oxidase (CYP88D6), 11-oxo-*β*-amyrin 30-oxidase (CYP72A154), are mainly expressed in roots and rhizomes, whereas no transcripts were observed in leaves^8,9,27^. Therefore, the screening of reference genes across different tissues or throughout different developmental stages, more importantly, the spatio (tissue)-temporal (developmental stage) interactions is of great importance for the study of gene functions in licorice.

Selection of reference genes, as of now, has been focused on a single factor/dimension, i.e., time (developmental stage) or space (tissue). However, the expression of genes is constantly under the influences of multiple factors/dimensions. Here, to better understand the spatio-temporal gene expression patterns in licorice, we investigated the expression profiles of 14 candidate reference genes in this study. Although Maroufi^28^ previously studied reference genes under the drought stresses in *G. glabra*, information concerning suitable reference genes is still lacking in licorice, especially for congeneric species, *G. uralensis* or G. *inflata*.

Based on previous research and our preliminary data, we hypothesized that internal references for spatio-temporal gene expression are different from the reference genes suited for individual conditions. To examine this hypothesis, we 1) evaluated the stability of 14 candidate genes, which were stably expressed housekeeping genes derived from our RNA-seq analysis; 2) selected optimal reference genes under the conditions of different tissues, developmental stages, and tissue × developmental stages, respectively; 3) compared the suitable reference genes under different experimental conditions and between the two congenic *Glycyrrhiza* species, and finally, we summarized the reference genes previously used within Leguminosae plants.

## Materials and methods

### Plant materials and growth conditions

**Figure 1:**
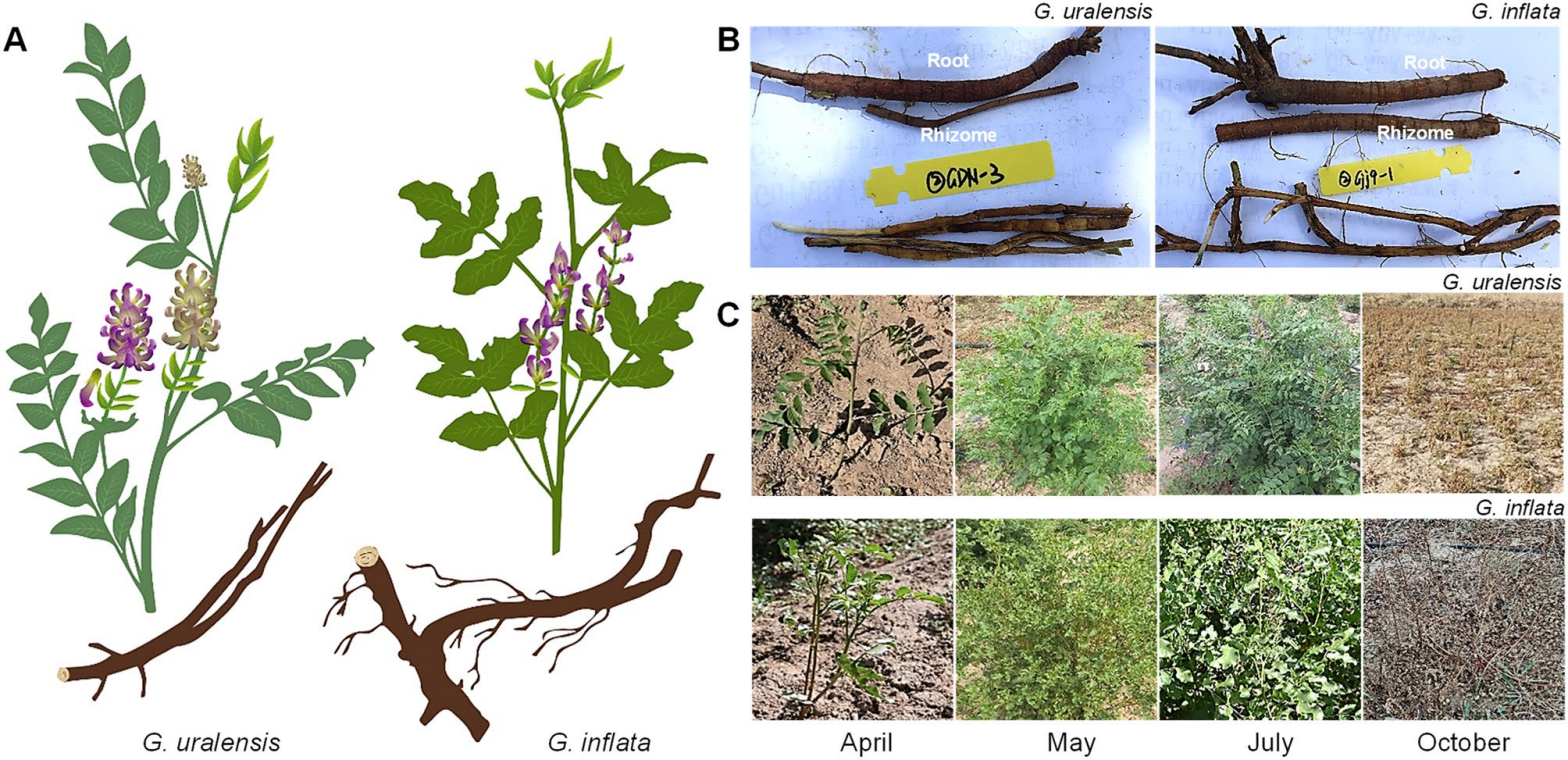
The morphology of different tissues under different developmental stages of *G. uralensis* and *G. inflata*. (A) schematic drawings of *G. uralensis* and *G. inflate,* respectively; (B) morphology of root and rhizome of *G. urelensis* and *G. inflata,* respectively; (C) *G. urelensis* and *G. inflate,* respectively, in different developmental stages, including Returning green stage (April), Rapid growth and flowering stage (May), Seed setting (July), and Senescence stage (October).

Two-year-old licorice plants (*G. uralensis* and *G. inflata*) were grown in the test field at the Northwest Biological Agricultural Center, Chinese Academy of Sciences (Ningxia, China). Roots, rhizomes and leaves were collected in April (returning green), May (rapid growth and flowering), July (seed setting), and October (aging) (Fig. 1). All samples were flash frozen in liquid nitrogen, shipped to Guangzhou in dry ice and stored at -80 ℃ for RNA extraction. All experiments were carried out with three biological replicates.

### RNA isolation and cDNA synthesis

Total RNA was isolated using HiPure Total RNA Mini Kit (Code No. R4151-03, Magen, China) according to the manufacturer’s instruction. The RNA quality and concentration were measured with agarose gel electrophoresis and spectrophotometer (NanoDrop 2000, Thermo, USA). Removal of genomic DNA contamination and first strand cDNA synthesis were performed using PrimeScript RT reagent Kit with gDNA Eraser (Code No. RR047A, Takara, Dalian, China).

### Candidate reference gene screening and primer design

A total of 14 candidate reference genes was included in this study, in which ten, (*Actin1* (*ACT*)*, Clathrin complex AP1* (*CAC*), *Cyclophilin* (*CYP*), *Heat-shock protein 40* (*DNAJ*), *Dehydration responsive element binding gene* (*DREB*), *Translation elongation factor1* (*EF1*), *Ras related protein* (*RAN*), *Translation initiation factor* (*TIF1*), *β-Tubulin* (*TUB*), *Ubiquitin-conjugating enzyme E2* (*UBC2*),) were selected in previous studies^28,29^, and the other four (*ATP binding-box transpoter 2* (*ABCC2*), *COP9 signal compex subunit 3* (*COPS3*), *Citrate synthase* (*CS*), *R3H domain protein 2* (*R3HDM2*) were selected from a RNA-seq dataset (SRA accession: PRJNA574093). RT-qPCR primers were designed using PrimerQuest Tool, INTEGRATED DNA TECHNOLOGIES (IDT) (https://sg.idtdna.com/Primerquest/Home/Index) based on the following parameters: Melting temperature (Tm) of 59–65 ℃; GC content of 45–55%; optimum length of 17–30 bp and amplicon length of 50–200 bp. All primers were synthesized by TSINGKE Company (Guangzhou, China). Detailed information listed in Table 1.

**Table 1:** Primers used in this study.

Gene	Description	Accession number	Primer sequence (5′–3′) Forward/reverse	Amplicon length (bp)	Tm (°C)	E (%)^a^
*ACT*	*Actin1*	MW119712	CCCACTCAACCCAAAGGC/TAACCCTCATAGATTGGCACAG	183	62.8	92.72
*CAC*	*Clathrin complex AP1*	MW116276	GAGTTTCAGCTTCCTCCTTGCA/TGATGGGGCTTTATCCTTTGG	126	63.4	116.84
*CYP*	*Cyclophilin*	MW119709	AAGACGGAGTGGCTGGACG/TCTTGCCGGAGCTGGACC	103	67	92.9
*DNAJ*	*Heat-shock protein 40*	MW116277	TGGTTGTCAAGGAACTGGTATG/ CACTGTGGGCAGCGGTCT	135	63.4	91.94
*DREB*	*Dehydration responsive element binding*	MW119710	GGTTGCTGAAATTCGGGAGC/ CATTGGGGAAGTTGAGGCG	139	64	97.83
*EF1*	*Translation elongation factor1*	MW116273	GACTGGTACAAGGGACCAAC/ AGACATCCTGCAATGGAAGC	101	63.1	90.42
*RAN*	*Ras related protein*	MW116274	ACAGAGCAGACGATGACTACGA/ CTGAGCCTTGATGACTTTGGA	185	63.2	91.22
*TIF1*	*Translation initiation factor*	MW122063	ACAACCGTTCAGGGATTGA/ GGGTCCTGAACAACTGTACC	98	62.2	77.95
*TUB*	*β-Tubulin*	MW119713	CCTTGAGCCAGGCACCAT/ GTCCTTTCGCCCAGTTGTT	113	63.6	86.97
*UBC2*	*Ubiquitin-conjugating enzyme E2*	MW116271	CTTCAACAAGACCCACCTGC/ ACGTGCCTCCATCCCATG	112	64.1	93.51
*ABCC2*	*ATP binding-box transporter 2*	MW116275	TGAGTCTTTCCAGGGCTTTATT/ATGGTGTTAAGGCGATGAGC	160	62.7	90.63
*COPS3*	*COP9 signal complex subunit 3*	MW119711	GGAAGCGCCAATACGAGG/ACAACAAGCACAGCAGAAGAAA	113	63.4	92.32
*CS*	*Citrate synthase*	MW116272	GCTCAGCCGTTGACCCAG/CACCACCAGGAAAAGCACC	93	64.2	107.58
*R3HDM2*	*R3H domain protein 2*	MW119714	GCTTTGGGTTCAATGGAGG/TCAGCAGAGTGCTGGGGTC	115	61.9	98.12

^a^Amplification efficiency.

### RT-qPCR analysis

The RT-qPCR were carried out in 384-well blocks using TB Green Premix Ex Taq II (Tli RNaseH Plus) (Code No. RR820D, Takara, Dalian, China) on LightCycler 480 (Roche, Switzerland) according to manufacturers’ instructions. Three technical repeats were carried out for each sample.

### Expression stability analysis of candidate reference genes

The slope of the standard curve of a cDNA tenfold dilution series was constructed to calculate the PCR amplification efficiency (E), and the E value was obtained according to the equation E = [10^(−1/slope)^ − 1] × 100. Expression stability of the 14 candidate reference genes were evaluated by four Microsoft Excel-based computational programs, geNorm^30^, NormFinder^31^, BestKeeper^32^ and Delta CT^33^. geNorm method ranks the expression stability by M value for each reference gene, and the smaller the M value, the more stable the gene. Based on NormFinder, the gene expression stability was calculated by the SV value. BestKeeper calculates the stability of candidate genes by the Pearson correlation coefficient (r), while the stability of the genes is evaluated by the pair-wise comparisons.

### Selection of optimal reference genes under different experimental conditions

The RefFinder, a comprehensive system to integrate the currently available major computational programs (geNorm, Normfinder, BestKeeper, and Delta Ct method) to compare and rank the stability of candidate reference genes, was used for the overall ranking of the candidate reference genes^34^. Based on the rankings from the Microsoft Excel-based computational programs, RefFinder assigns an appropriate weight to an individual gene and calculates the geometric mean of their weights for the overall final ranking.

### Comparison of the suitable reference genes under different experimental conditions and between the two congenic *Glycyrrhiza* species

The top three most suitable reference genes selected by RefFinder under the conditions of different tissues, developmental stages, and tissue × developmental stages were compared; and the suitable reference genes under the same experimental condition between the two congenic *Glycyrrhiza* species were also analyzed. The results were visualized by Venn Diagrams, and it was plotted using the OmicShare tools, a free online platform for data analysis (www.omicshare.com/tools).

### Validation of recommended reference genes

To confirm the suitability of the reference genes recommended in the present study, we measured the differential expression of a specific licorice gene with a known expression profile under different tissues. Licorice *β-amyrin synthase* (*β-AS*, GenBank Accession Number: FJ627179), is a key gene in glycyrrhizin biosynthesis and mainly expressed in root organs^8, 27^, we thus chose to validate the reliability of the selected reference genes in different treatment conditions. The primers of *β-AS* used for RT-qPCR were listed in Table 1. The expression level of *β-AS* was analyzed by seven normalization ways, including the most stable, the top two most stable, the top three most stable, the least stable, the top two least stable, the top three least stable, and all the candidate reference genes. One way ANOVA was carried out to evaluate the expression level of *β-AS* under different normalization conditions (SPSS statistics 22.0 software, IBM, United States).

### Survey of the reference genes used within Leguminosae plants

Reference genes reported previously in Leguminosae species was selected by searching “NCBI” or “Web of Science” with the key words “Leguminosae” and “reference gene”. The top three most suitable reference gene was considered to calculate the recommendation frequency for developmental stages and tissues, respectively, among Leguminosae species.

## Results

### Primer specificity and RT-qPCR amplification efficiency

The specificity of the primers for all 14 candidate reference genes (*ACT*, *CAC*, *CYP*, *DNAJ*, *DREB*, *EF1*, *RAN*, *TIF1*, *TUB*, *UBC2*, *ABCC2*, *COPS3*, *CS*, *R3HDM2*) were determined by a single PCR product of expected size, and further confirmed by a single peak in the melting-curve analysis. The efficiency of the PCR amplification (E) was calculated from the standard curve by making a dilution series with mixed samples. The E value of the reported reference genes ranged from 67.28% (*CAC*) to 116.84% (*CYP*) and correlation coefficients varied from 0.9526 (*CAC*) to 0.9976 (*CYP*) (Table 1). Three of them, *CAC*, *TIF1* and *TUB*, presented unmatched E values which did not meet the requirements (90%—110%) for RT-qPCR analysis. The E value of the selected candidate reference genes from RNA-Seq data ranged from 90.63% (*CAC*) to 107.58% (*CYP*) and correlation coefficients varied from 0.9887 (*CS*) to 0.9963 (*COPS3*) (Table 1), and all these primers are suitable for RT-qPCR analysis^32^.

### Expression profiling of candidate reference genes

**Figure 2:**
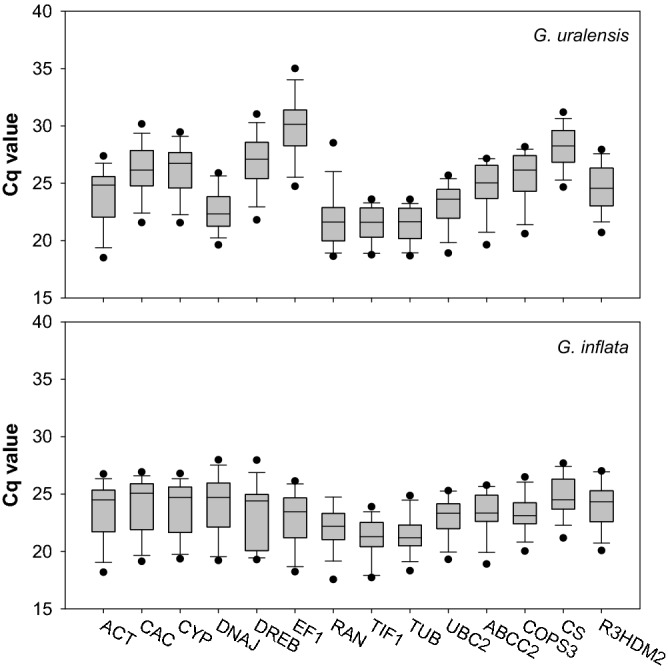
Distribution of threshold cycle (Cq) values for candidate reference genes. Distribution of threshold cycle (Cq) values of *G. uralensis* and *G. inflata* in all samples. Boxes: the interquartile range; lines across the boxes: median; lines above and below the boxes: the maximum and minimum values; black dots: outliers.

The expression levels of the candidate reference genes were determined as quantification cycle (Cq) values, and the transcripts of these genes showed different levels of abundance in *G. uralensis* and *G. inflata* (Tables S1 and S2). The mean Cq values of the genes ranged from 20—27, with the majority lying between 23 and 26 across all tested samples in *G. uralensis*, and the mean Cq values of the genes ranged from 19—25, with the majority lying between 22 and 25 across all tested samples in *G. inflata* (Fig. 2, Tables S3 and S4). So the expression level of these candidate genes were much higher in tested samples in *G. inflata* than in *G. uralensis*, and they were more stable in *G. inflata* than in *G. uralensis*.

*TIF1* had the lowest Cq both in *G. uralensis* (mean Ct of 20.30) and *G. inflata* (mean Cq of 19.94), indicating the highest expression level of *TIF1* in the two species, while *EF1* (mean Cq of 27.50) or *DREB* (mean Cq of 25.56) was expressed at low levels in *G. uralensis* or *G. inflata*, respectively (Tables S3 and S4).

*CAC* showed the least gene expression variation both in *G. uralensis* (coefficient of variation (CV) of 6.74%) and *G. inflata* (CV of 2.92%), while surprisingly, a commonly used reference gene, *ACT*, was the most variable across all samples (CV of 10.45%) in *G. uralensis* (Table S3), and *TIF1* (9.60%) was the most variable across all samples in *G. inflata* (Table S4).

### Expression stability of candidate reference genes

The expression profiles of the 14 candidate reference genes in *G. uralensis* and *G. inflata* roots, rhizomes, and leaves across all experiments in this study were analyzed using geNorm, NormFinder, BestKeeper, Delta CT, and RefFinder (Tables 2 and 3).

**Table 2:** Stability of candidate reference genes under different conditions in *G. uralensis.*

Candidate genes	geNorm		NormFinder		BestKeeper		∆Ct method		RefFinder		Comprehensive recommendation
	Stability	Rank	Stability	Rank	Stability	Rank	Stability	Rank	Stability	Rank	
**Developmental stages**											
*ACT*	0.331	7	0.245	5	0.23	4	0.49	6	5.38	7	*CAC*, *CYP*, *ABCC2*
*CAC*	0.077	1	0.128	1	0.18	3	0.43	1	1.32	1	
*CYP*	0.077	1	0.199	3	0.24	5	0.45	4	2.78	2	
*DNAJ*	0.359	9	0.349	9	0.12	1	0.51	9	5.20	6	
*DREB*	0.295	5	0.203	4	0.39	11	0.45	3	5.07	5	
*EF1*	0.514	13	0.778	14	0.86	14	0.82	13	13.49	14	
*RAN*	0.346	8	0.281	7	0.24	6	0.5	8	7.20	9	
*TIF1*	0.558	14	0.771	13	0.37	10	0.82	14	12.63	13	
*TUB*	0.392	10	0.477	10	0.62	13	0.59	10	10.68	10	
*UBC2*	0.468	12	0.645	12	0.31	8	0.71	12	10.84	11	
*ABCC2*	0.264	4	0.191	2	0.36	9	0.44	2	3.46	3	
*COPS3*	0.146	3	0.248	6	0.17	2	0.47	5	3.66	4	
*CS*	0.424	11	0.551	11	0.45	12	0.65	11	11.24	12	
*R3HDM2*	0.311	6	0.281	8	0.26	7	0.49	7	6.96	8	
**Tissues**											
*ACT*	0.322	6	0.503	7	1.69	10	0.73	7	7.36	8	*R3HDM2*, *CAC*, *TUB*
*CAC*	0.104	1	0.044	1	1.39	6	0.60	3	2.06	2	
*CYP*	0.420	9	0.656	10	1.71	11	0.82	10	9.97	13	
*DNAJ*	0.180	4	0.088	4	1.32	5	0.61	4	4.23	4	
*DREB*	0.360	7	0.558	8	1.77	12	0.71	6	7.97	9	
*EF1*	0.476	10	0.978	12	2.06	14	1.03	12	11.92	14	
*RAN*	0.538	11	0.455	6	1.18	2	0.76	8	5.70	5	
*TIF1*	0.716	13	1.101	13	1.21	3	1.17	13	9.01	11	
*TUB*	0.154	3	0.064	3	1.41	8	0.58	2	3.46	3	
*UBC2*	0.624	12	0.814	11	1.25	4	0.96	11	8.73	10	
*ABCC2*	0.254	5	0.339	5	1.63	9	0.63	5	5.79	6	
*COPS3*	0.808	14	1.310	14	0.76	1	1.36	14	7.24	7	
*CS*	0.389	8	0.650	9	1.82	13	0.77	9	9.58	12	
*R3HDM2*	0.104	1	0.052	2	1.4	7	0.57	1	1.93	1	
**Tissues × developmental stages**											
*ACT*	0.614	8	0.63	6	1.89	13	0.94	7	8.13	11	*CAC*, *R3HDM2*, *DNAJ*
*CAC*	0.364	1	0.273	2	1.36	5	0.76	1	1.78	1	
*CYP*	0.562	7	0.685	10	1.56	9	0.95	9	8.68	13	
*DNAJ*	0.516	6	0.26	1	1.47	6	0.77	3	3.22	3	
*DREB*	0.475	5	0.469	4	1.7	11	0.81	4	5.45	5	
*EF1*	0.975	14	1.206	14	2.06	14	1.33	14	14.00	14	
*RAN*	0.715	10	0.659	8	1.32	4	0.97	10	7.52	9	
*TIF1*	0.855	12	1.107	12	1.3	2	1.26	12	8.49	12	
*TUB*	0.663	9	0.643	7	1.54	8	0.95	8	7.97	10	
*UBC2*	0.781	11	0.858	11	1.3	3	1.07	11	7.18	8	
*ABCC2*	0.402	4	0.497	5	1.59	10	0.83	5	5.23	4	
*COPS3*	0.915	13	1.158	13	0.87	1	1.31	13	6.85	6	
*CS*	0.456	5	0.667	9	1.72	12	0.92	6	7.14	7	
*R3HDM2*	0.364	1	0.297	3	1.47	7	0.77	2	2.55	2

**Table 3:** Stability of candidate reference genes under different conditions in *G. inflata*.

Candidate genes	geNorm		NormFinder		BestKeeper		∆Ct method		RefFinder		Comprehensive recommendation
	Stability	Rank	Stability	Rank	Stability	Rank	Stability	Rank	Stability	Rank	
**Developmental stages**											
*ACT*	0.855	14	1.468	14	1.2	14	1.55	14	14.00	14	*RAN*, *COPS3*, *CS*
*CAC*	0.424	8	0.351	7	0.59	11	0.74	7	8.10	7	
*CYP*	0.680	12	0.987	13	0.44	7	1.11	12	10.70	12	
*DNAJ*	0.501	9	0.517	8	0.56	10	0.81	8	8.71	8	
*DREB*	0.242	5	0.263	4	0.29	4	0.65	4	4.47	4	
*EF1*	0.280	6	0.336	6	0.29	5	0.69	6	5.42	6	
*RAN*	0.109	1	0.097	1	0.22	2	0.60	1	1.19	1	
*TIF1*	0.740	13	0.987	12	0.71	12	1.13	13	12.49	13	
*TUB*	0.359	7	0.777	11	0.55	9	0.94	11	9.34	10	
*UBC2*	0.617	11	0.690	9	0.79	13	0.92	10	10.65	11	
*ABCC2*	0.203	4	0.281	5	0.39	6	0.67	5	4.95	5	
*COPS3*	0.109	1	0.226	2	0.17	1	0.62	2	1.41	2	
*CS*	0.116	3	0.226	3	0.24	3	0.63	3	3.00	3	
*R3HDM2*	0.564	10	0.696	10	0.48	8	0.91	9	9.21	9	
**Tissues**											
*ACT*	0.103	4	0.366	6	0.61	9	0.61	6	6.00	8	*COPS3*, *R3HDM2*, *DREB*
*CAC*	0.432	10	0.470	9	0.24	1	0.71	10	5.48	6	
*CYP*	0.658	13	1.126	13	1.12	14	1.17	13	13.24	14	
*DNAJ*	0.339	8	0.377	7	0.40	3	0.66	7	5.86	7	
*DREB*	0.070	1	0.237	4	0.56	8	0.55	4	3.36	3	
*EF1*	0.582	12	0.892	12	1.04	13	1.03	12	12.24	12	
*RAN*	0.392	9	0.381	8	0.46	5	0.67	8	7.33	10	
*TIF1*	0.759	14	1.333	14	0.77	11	1.36	14	13.18	13	
*TUB*	0.086	3	0.311	5	0.62	10	0.58	5	5.23	5	
*UBC2*	0.507	11	0.834	11	0.39	2	0.95	11	7.18	9	
*ABCC2*	0.185	6	0.101	2	0.41	4	0.55	3	3.46	4	
*COPS3*	0.124	5	0.034	1	0.52	6	0.53	1	2.34	1	
*CS*	0.237	7	0.512	10	0.80	12	0.70	9	9.32	11	
*R3HDM2*	0.070	1	0.150	3	0.55	7	0.54	2	2.55	2	
**Tissues × developmental stages**											
*ACT*	1.27	14	1.465	13	1.35	14	1.68	14	13.74	14	*COPS3*, *DREB*, *ABCC2*
*CAC*	0.703	5	0.54	3	0.75	4	1.06	3	3.66	4	
*CYP*	1.123	12	1.418	12	1.11	10	1.64	12	11.47	12	
*DNAJ*	0.783	6	0.603	5	0.88	8	1.1	5	5.89	6	
*DREB*	0.466	1	0.495	2	0.67	3	1.03	2	1.86	2	
*EF1*	0.988	10	0.970	9	1.18	13	1.32	9	10.13	10	
*RAN*	0.835	7	0.717	7	0.83	7	1.16	7	7.00	7	
*TIF1*	1.201	13	1.470	14	1.11	11	1.68	13	12.70	13	
*TUB*	1.044	11	1.129	11	1.16	12	1.42	11	11.24	11	
*UBC2*	0.930	9	1.027	10	0.81	6	1.34	10	8.57	9	
*ABCC2*	0.466	1	0.680	6	0.63	1	1.12	6	2.45	3	
*COPS3*	0.611	4	0.246	1	0.65	2	0.95	1	1.68	1	
*CS*	0.532	3	0.569	4	0.8	5	1.06	4	3.94	5	
*R3HDM2*	0.876	8	0.816	8	1.02	9	1.21	8	8.24	8	

For different developmental stages, four key growth periods of licorice were selected and measured, including Returning green stage in April; Rapid growth and flowering stage in May; Seed setting stage in July; and Senescence stage in October (Fig. 1). In *G. uralensis*, *CAC*, *CYP*, *COPS3* were the most stable reference genes recommended by geNorm, whereas *CAC*, *ABCC2*, *CYP* by NormFinder, *DNAJ*, *COPS3*, *CAC* by the BestKeeper, and *CAC*, *ABCC2*, *DREB* by Delta CT (Table 2). In *G. inflata*, the top three most stable candidate reference genes were *RAN*, *COPS3*, *CS* identified by all four methods, geNorm, NormFinder, BestKeeper and Delta CT (Table 3).

For different tissues, three tissues (the roots, rhizomes and leaves) were tested (Fig. 1). In *G. uralensis*, the top three most stable reference genes recommended by geNorm and NormFinder were *CAC*, *R3HDM2* and *TUB*, while *COPS3*, *RAN* and *TIF1* by BestKeeper, *R3HDM2*, *TUB* and *CAC* by Delta CT (Table 2). In *G. inflata*, *DREB*, *R3HDM2* and *TUB* were the most stable reference genes recommended by geNorm, *COPS3*, *ABCC2*, *R3HDM2* by NormFinder, *CAC*, *UBC2* and *DNAJ* by BestKeeper, *COPS3*, *R3HDM2* and *ABCC2* by Delta CT (Table 3).

Because the growth, development, and metabolite accumulation of all the living organisms are certainly influenced by multiple factors, therefore, we also studied the optimal reference genes under the spatial–temporal interaction conditions in both *G. uralensis* and G. *inflata*. In *G. uralensis*, the top three most stable candidate reference genes were *CAC*, *R3HDM2*, *ABCC2* identified by geNorm, *DNAJ*, *CAC*, *R3HDM2* by NormFinder, *COPS3*, *TIF1*, *UBC2* by BestKeeper, and *CAC*, *R3HDM2*, *DNAJ* by Delta CT (Table 2). In *G. inflata*, *DREB*, *ABCC2*and *CS* were the most stable reference genes recommended by geNorm, *COPS3*, *DREB* and *CAC* by NormFinder and Delta CT, *ABCC2*, *COPS3* and *DREB* by BestKeeper (Table 3).

### Selection of optimal reference genes under different experimental conditions

**Figure 3:**
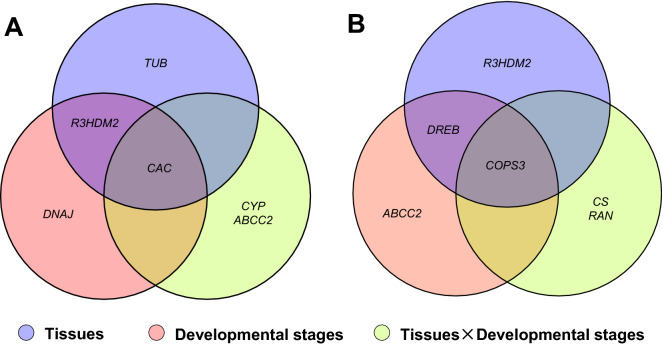
Stability of candidate reference genes under different conditions in *G.urelensis* and *G. inflata*. The top three most suited reference genes under different experimental conditions in *G. uralensis* (A) and *G. inflata* (B).

Based on RefFinder, a web-based software, comprehensive ranking of reference genes integrating all four software was obtained (Tables 2 and 3). At different experimental stages, *CAC*, *CYP*, *ABCC2* were identified as the top three most stable reference genes in the *G. uralensis* (Fig. 3A, Table 2), while *RAN*, *COPS3*, *CS* were identified in *G. inflata* (Fig. 3B, Table 3). For the different tissues, *R3HDM2*, *CAC*, *TUB* were identified as the most stable reference genes in the *G. uralensis* (Fig. 3A, Table 2), while *COPS3*, *R3HDM2*, *DREB* were identified in the *G. inflata* (Fig. 3B, Table 3). Under the spatial–temporal interaction conditions in *G. uralensis*, *CAC*, *R3HDM2*, *DNAJ* were identified as the most stable reference genes in the *G. uralensis* (Fig. 3A, Table 2), while *COPS3*, *DREB*, *ABCC2* were identified in the *G. inflata* (Fig. 3B, Table 3).

**Figure 4:**
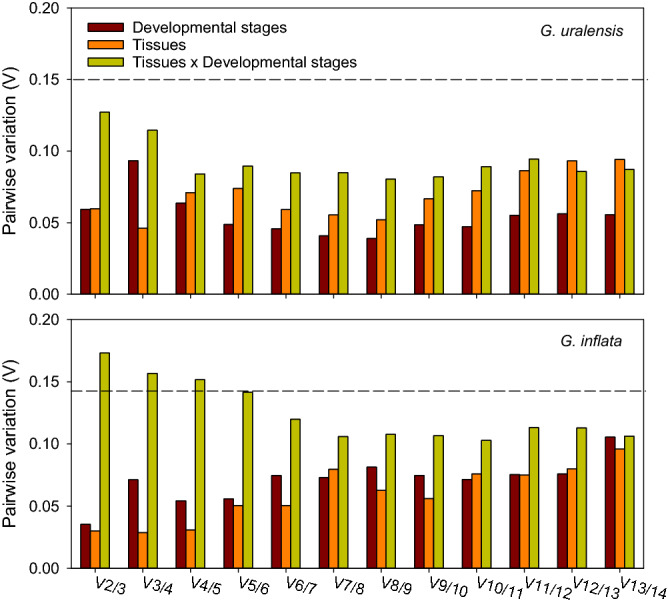
Pairwise variation analysis of the candidate reference genes. Pairwise variation analysis in *G. uralensis* and *G. inflata*, respectively, by geNorm procedure. The pairwise variation (V_n_/V_n+1_) was calculated with the normalization factors NF_n_ and NF_n+1_ to determine the optimal number of reference genes required for RT-qPCR data normalization under different conditions.

The optimal number of reference genes under each experimental conditions required for reliable normalization in two species were predicted by geNorm software with the V values (cutoff = 0.15). When pairwise variations Vn/n + 1 < 0.15, it means that an addition reference gene (n + 1) is not necessary. For all the experimental conditions in *G. uralensis* the first V-value less than 0.15 occurred at V2/3, suggesting that two reference genes were adequate to correctly normalize gene expression. But for spatial–temporal interaction conditions in the *G. inflata*, more than two reference genes was necessary suggested by V-value for accurate normalization, the first V-value less than 0.15 occurred at V4/5 (Fig. 4).

### Comparison of the suitable reference genes under different experimental conditions and between the two congenic *Glycyrrhiza* species

The summary of the top three most suitable reference genes under all experimental condition showed that seven genes appeared in the top three list in *G. uralensis*, among which *CAC* and *R3HDM2* showed the highest recommended frequency (33.33% and 22.22%, respectively) (Fig. 3A). In *G. inflata*, seven genes were also appeared in the top three lists, and COPS3 and *DREB* presented the highest recommended frequency, with the frequency of 33.33% and 22.22%, respectively (Fig. 3B).

Comparison of the suitable reference genes under different experimental conditions showed only one of the top three candidates was shared between the individual factors and their interactions, specifically, *CAC* in *G. uralensis* and *COPS3* in *G. inflata*. Therefore, *CAC* was the most stable reference gene in *G. uralensis* under all the experimental conditions tested, while *COPS3* was the most stable in *G. inflata*.

For the comparison of the suitable reference genes between the two congenic *Glycyrrhiza* species, we found *R3HDM2* was the only suitable reference gene shared between *G. uralensis* and *G. inflata* in different tissues, and no consistent reference gene was found under different developmental stage and tissue and developmental stage interactions between the two congenic *Glycyrrhiza* species. So the optimal reference genes for different species are variable, even for the two proximal species in the same genus.

### Validation of recommended reference genes

**Figure 5:**
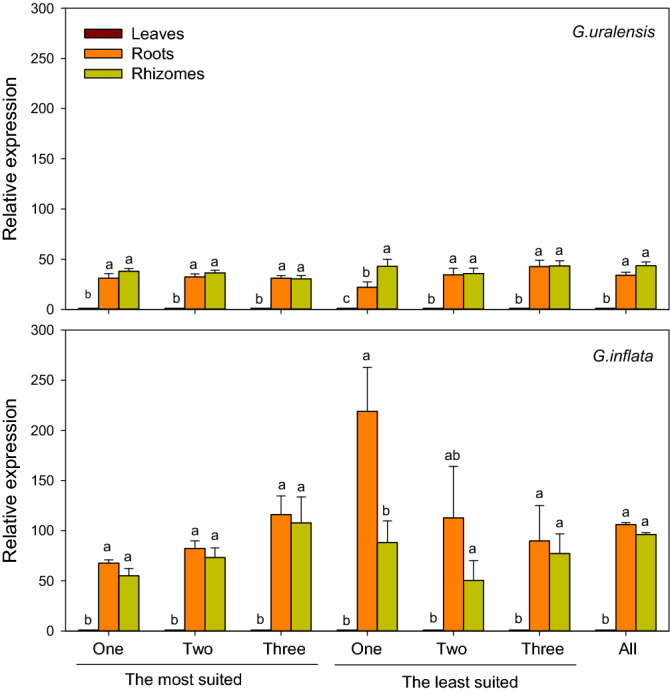
Validation of the recommended reference genes. Expression profiles of *β-AS* gene among different tissues were investigated using seven different normalization factors. The most suited, the top two most suited, the top three most suited, the least suited, the top two least suited, the top three least suited, and all the candidate reference genes. Bars represent the means ± standard error of biological replications. Different letters indicate significant differences (*P* < 0.05).

A root and rhizome-specific gene, *β-AS*, a key gene in glychrizin biosynthesis was used to validate the selected reference genes. To validate the reference geneto study expressin pattern in different tissues, expression of *β-AS* was normalized to both the most and the least stablecandidate reference genes, both in *G.uralensis* and *G. inflata*. When the recommended reference genes were used, the expression levels of *β-AS* in the roots and the rhizomes were similar and both high, while its expression was significantly reduced in the leaf. However, when normalized to the least suited reference genes, the expression pattern of *β-AS* changed, or the ratio of expression levels between roots/rhizomes and leaves were significantly enlarged or narrowed (Fig. 5). Unstable reference genes really confuse the results.

### Survey of the reference genes used within Leguminosae plants

Reference gene selection has been reported in 12 Leguminosae species (*Arachis hypogaea* L., *Cassia obtusifolia* L., *Cicer arietinum* L., *Cyamopsis tetragonoloba* L.Taub, *Eremosparton songoricum* (Litv.) Vass., *Glycine max* (L.) Merr., *Hedysarum coronarium* L., *Lens culinaris* Medic., *Lupinus angustifolius* L., *Medicago sativa* L., *Phaseolus vulgaris* Linn., *Vigna angularis* (Willd.) Ohwi et Ohashi) under different experimental conditions, including different tissues or different experimental stages. In this study, we added *G. uralensis* and *G. inflata* to this list.

**Figure 6:**
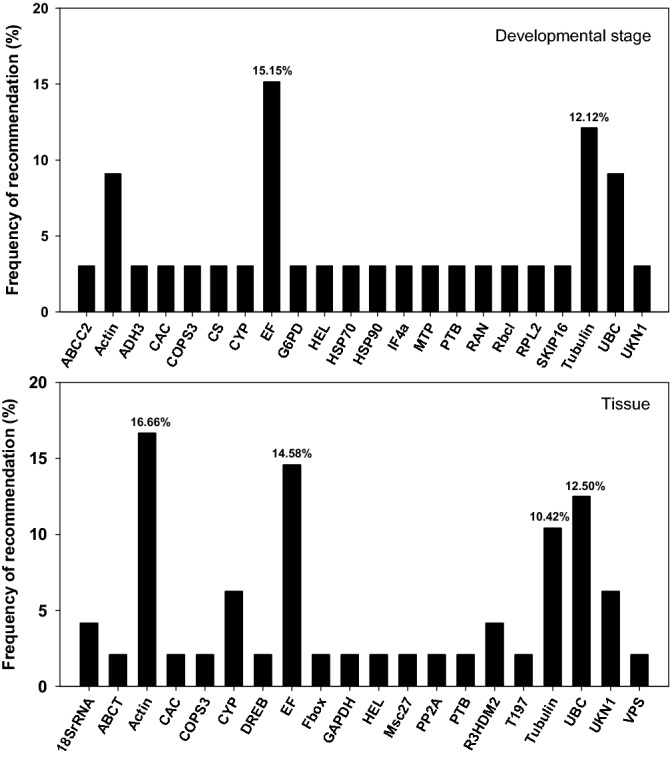
Frequency of reference genes recommended for RT-qPCR analysis under different conditions in Leguminosae plants. Here we surveyed the frequency of each reference gene recommended for different developmental stages and different tissues among the twelve Leguminosae species. Reference genes (top three) recommended for each species under different developmental stages and tissues are detailed in Table S5.

A total of ten species (including *G. uralensis* and *G. inflata*) had been studied under different developmental stages, among the 22 reference genes recommended, *eukaryotic elongation factor* (*EF*, *EF1α*, and *ELF1B*) and tubulin (*TUA1*, *TUA2*, *TUA5*, and *TUB*) were the most choices, and the frequency of recommendation in the 10 species were 15.15% and 12.12%, respectively (Fig. 6). For the different tissues, a total of twelve species had been studied. Among the 20 reference genes recommended, *ACT*, *EF*, and *UBQ* performed particularly well, and they presented recommended frequency of 16.66%, 14.58% and 12.50%, respectively (Fig. 6).

## Discussion

Licorice are herbaceous perennial plants with great medical and ecological values. Licorice root extracts have been proved to have anti-carcinogenic^2,3^, anti-inflammatory, anti-fungal, anti-piroplasmic and cytotoxic activities^4^. More recently, glycyrrhizin, the most important bioactive triterpenoid saponin in licorice roots, is under the consideration for treating COVID-19 infection caused respiratory syndrome^5^. Accumulation of its many bioactive compounds is spatio-temporal dependent. To study the functional genes involved in development and biosynthesis of these bioactive compounds, spatio-temporal expression pattern of these genes provides an important piece of information. Here we report a set of reference genes for RT-qPCR analysis to study spatio-temporal expression pattern of genes in two congenic licorice species, *G. uralensis* and *G. inflata*.

### Candidates screening and optimal reference genes selection

In this study, *CAC* and *COPS3* was one of the top candidates for all three experimental conditions tested in *G. uralensis* and *G. inflata,* respectively. *COPS3* is a new candidate of reference gene screened from RNA-seq, and it was proved to be the most stable gene under different developmental stages in *G. inflata*. This result supports the idea that it is feasible to screen reference genes through RNA-seq dataset^35^. The current and emerging RNA-seq data may provide a bigger and even more reliable pool to select candidate reference genes other than the traditional housekeeping genes. Such discoveries are of great significance and should enable greater accuracy of normalization, particularly across diverse plant organs and in other experimental conditions where traditional housekeeping genes display variability in expression.

### The suitable reference genes for spatio-temporal gene expression is different from that for individual conditions

Our results showed that different reference genes were selected to study spatial, temporal expression as well as spatial–temporal expression patterns in both species. The most stable reference genes varied in different experiments. In *G. uralensis*, the top three most stable reference genes across different tissues were *R3HDM2*, *CAC* and *TUB*, while *CAC*, *CYP* and *ABCC2* were most suited for different developmental stages. Similarly, in *G. inflata, COPS3*, *R3HDM2* and *DREB* were selected for tissues, while *RAN*, *COPS3* and *CS* were recommended for developmental stages (Tables 2 and 3). In addition, the optimal reference genes under the condition of two-factor interaction (developmental stages × tissues) are also different from those under single factor conditions. For the tissue-developmental stage interactions, *CAC*, *R3HDM2* and *DNAJ* were the most suited reference genes in *G. uralensis*, while *COPS3*, *DREB* and *ABCC2* in *G. inflata*. Only one of the top three candidates was shared between the individual factors and their interactions, specifically, *CAC* in *G. uralensis* and *COPS3* in *G. inflata* (Fig. 3). Because the expression of genes is constantly under the influences of multiple factors/dimensions, so it is essential for gene function analysis to investigate gene expression under the interacting factors. Our results in this study illustrated that the optimal reference gene for spatio-temporal gene expression is different from that for individual conditions, so every gene expression analysis should begin with validation of reference genes in a given sample set under specific experiment conditions, either under single factor or under multiple factors interacting conditions.

The validation of selected reference genes was done by normalizing the expression of *β-AS*, a key gene involved in glycyrrhizin biosynthesis, and its expression has been proven to be mainly in roots and rhizomes^8,9^. Generally, the expression pattern of *β-AS* should not be affected by reference gene selection, because the M values of the 14 candidate genes selected in our study were all below 1.5 by geNorm. However, our results showed that the expression pattern of *β-AS* was quite different when using the unstable reference genes for homogenization compared with the stable reference genes (Fig. 5). Therefore, our results showed that unstable reference genes would confuse the expression pattern while the stable reference genes gave reliable results, and the optimal reference genes screened in this study are reliable.

### The optimal reference genes within Leguminosae species are drastically different

In this study, we also summarized the validated reference genes for different development stages in Leguminosae (including *G. uralensis* and *G. inflata* tested in this study). From the results of our survey, we found *EF1*, *TUB* and *UBQ* are commonly used housekeeping genes, which have been identified as the most suitable ones at different developmental stages in several species. *EF* had been selected as the most suitable reference gene at different developmental stages in *E. songoricum*^36^, *G. max*^37^, and *C. arietinum*^38^. *TUB* was the optimal reference gene at different experimental stages in *E. songoricum*^36^, *G. max*^37^, and *H. coronarium*^39^. While *UBQ* was validated as the most stable reference gene at different developmental stages in *C. tetragonoloba*^40^, *H. coronarium*^39^, *L. angustifolius*^41^. For the different tissues in Leguminosae, the most stable reference gene candidates were *ACT*, *EF* and *UBQ*. Among them, *ACT* was recommended as a stable reference gene in different tissues of *C. tetragonoloba*^40^, *E. songoricum*^36^, *G. max*^37,42^, *P. vulgaris*^43^. *EF* was recommended in *C. obtusifolia*^44^, *G. max*^37^, *M. sativa*^45^, and *UBQ* was recommended in *G. inflata* (Table 3), *E. songoricum* ^36^, *G. max*^37^, and *L. angustifolius*^41^. So, the optimal reference genes for different species in the same family are variable, even for the two proximal species in the same genus (*G. uralensis* and *G. inflata*). We found *CAC* was the most stable reference gene when all factors taken account in *G. uralensis*, and *COPS3* was the optimal reference gene with the highest recommended frequency in *G. inflata* (Fig. 3). However, *EF1* and *TIF1* in *G. uralensis*, and *ACT* and *TIF1 G. inflata* were the most unstable reference genes respectively, and it has been proved that it will cause false results using the unstable reference gene for expression normalization (Fig. 5). Among them, *EF1* and *ACT* are commonly used housekeeping gene and have been identified as the most suitable reference genes in several studies^40,43,46^. So our results indicated that it is always necessary to validate reference genes for reliable gene expression analysis. The summary and analysis of the reported legume reference genes will serves as a guide for the subsequent selection of reference genes in Leguminosae plants.

### Summary and perspectives

In this study, we evaluated the expression of 14 candidate reference genes across different tissues (root, rhizome, leaf) at various developmental stages (returning green, April; rapid growth and flowering, May; seed setting, July; and senescence stage, October) in the two congeneric medicinal plants, *G. uralensis* and G. *inflate*, respectively. Based on previous research and our preliminary data, we hypothesized that internal references for spatio-temporal gene expression are different from the reference genes suited for individual factors. In *G. uralensis*, the top three most stable reference genes across different tissues were *R3HDM2*, *CAC* and *TUB*, while *CAC*, *CYP* and *ABCC2* were most suited for different developmental stages. *CAC* is the only candidate recommended for both biotic factors, which is reflected in the stability ranking for the spatio (tissue)-temporal (developmental stage) interactions (*CAC*, *R3HDM2* and *DNAJ*). Similarly, in *G. inflata, COPS3*, *R3HDM2* and *DREB* were selected for tissues, while *RAN*, *COPS3* and *CS* were recommended for developmental stages. For the tissue-developmental stage interactions, *COPS3*, *DREB* and *ABCC2* were the most suited reference genes. In both species, only one of the top three candidates was shared between the individual factors and their interactions, specifically, *CAC* in *G. uralensis* and *COPS3* in *G. inflata*, which supports our overarching hypothesis.

In addition, we also documented the reference genes that have been used in RT-qPCR analyses among 12 different Leguminosae plants under the same biotic conditions with current study, i.e., tissue and/or developmental stage. Among the 115 genes have been tested, even the routinely used reference genes showed variable expressions under different experimental conditions. Therefore, to avoid the misinterpretation of RT-qPCR results, a thorough evaluation of reference genes is strongly recommended. More importantly, given that biosynthesis of bioactive compounds is typically spatio-temporal dependent, the selection of suitable reference genes should follow suit. Based on previous studies and our current results, we concluded (1) transcriptome is a rich reservoir for selecting stably expressed candidate reference genes, (2) customized design, especially the interaction among the experimental conditions, is warranted for searching suitable reference genes in any given species, and (3) without validation study, gene(s), including housekeeping genes, could lead to ambiguous results, especially in non-model species. Finally, spatio-temporal selection of reference genes not only lays the foundation for functional genomics research in *Glycyrrhiza*, but also facilitates these traditional medicinal herbs to reach/maximize their pharmaceutical potential.

## Supplementary information

Supplementary Information 1.

## Data Availability

Data will be available upon request. RNA-seq datasets used in this study can be found in online repositories. The names of the repository/repositories and accession number(s) can be found below: https://www.ncbi.nlm.nih.gov/, PRJNA574093.

## Abbreviations

ABCC2: ATP binding-box transporter 2
ACT: Actin1 gene
CAC: Clathrin complex AP1
COPS3: COP9 signal complex subunit 3
CS: Citrate synthase
CYP: Cyclophilin
CYP88D6: β-Amyrin 11-oxidase
DNAJ: Heat-shock protein 40
DREB: Dehydration responsive element binding gene
E: The efficiency of the PCR amplification
EF1: Translation elongation factor1
MIQE: Minimum information for publication of quantitative real-time PCR experiments
RT-qPCR: Reverse transcription quantitative real-time PCR
R3HDM2: R3H domain protein 2
RAN: Ras related protein
TIF1: Translation initiation factor
TUB: β-Tubulin
UBC2: Ubiquitin-conjugating enzyme E2
β-AS: β-Amyrin synthase
