# Supplementary material for: Spatio-temporal selection of reference genes in the two congeneric species of Glycyrrhiza
Source: Sci Rep. 2021 Mar 2;11:1122. doi: 10.1038/s41598-020-79298-8 (PMC7925658; doi:10.1038/s41598-020-79298-8)
Supplement: Supplementary file 1 — Supplementary Information 1. [file 41598_2020_79298_MOESM1_ESM.docx]

**Spatio-temporal selection of reference genes in the two congeneric species of *Glycyrrhiza***

Yuping Li^1, 2, §^, Xiaoju Liang^1, 3, §^, Xuguo Zhou^4^, Yu An^5^, Ming Li^5^, Ling Yuan^1, 2, 6^, Yongqing Li^1, 2,^ ^*^, Ying Wang^1, 2, 7, *^

^1^ Key Laboratory of South China Agricultural Plant Molecular Analysis and Genetic Improvement & Guangdong Provincial Key Laboratory of Applied Botany, South China Botanical Garden, Chinese Academy of Sciences, Guangzhou 510650, China.

^2^ Center of Economic Botany, Core Botanical Gardens, Chinese Academy of Sciences, Guangzhou, 510650, China.

^3^ University of Chinese Academy of Sciences, Beijing 100049, China.

^4^ Department of Entomology, University of Kentucky, Lexington, KY 40546, USA.

^5^ Institute of Desertification Control, Ningxia Academy of Agriculture and Forestry Sciences, Yinchuan 750002, China.

^6^ Department of Plant and Soil Sciences, University of Kentucky, Lexington, KY 40546, USA.

^7^ Gannan Normal University, Ganzhou, Jiangxi, 341000, P.R. China.

-------------------------------------------------------------------------------------------------------

^§^ These authors contributed equally

^*^ For correspondence

yingwang@scib.ac.cn (Y. Wang)

liyongqing@scbg.ac.cn (Y. Li)

**Table S1. Cq values under different experimental conditions in *G. uralensis*.**

|  | **Conditions** | ***ACT*** | ***CAC*** | ***CYP*** | ***DNAJ*** | ***DREB*** | ***EF1*** | ***RAN*** | ***TIF1*** | ***TUB*** | ***UBC2*** | ***ABCC2*** | ***COPS3*** | ***CS*** | ***R3HDM2*** |
| --- | --- | --- | --- | --- | --- | --- | --- | --- | --- | --- | --- | --- | --- | --- | --- |
| Returning green stage (April) | Taproot_1 | 22.37 | 25.43 | 23.18 | 22.08 | 26.11 | 27.37 | 25.53 | 18.51 | 25.37 | 20.27 | 21.98 | 25.71 | 24.94 | 24.75 |
|  | Taproot_2 | 25.24 | 28.04 | 25.47 | 24.65 | 28.56 | 30.17 | 27.81 | 21.57 | 27.14 | 23.22 | 24.82 | 25.62 | 27.14 | 26.66 |
|  | Taproot_3 | 25.54 | 27.81 | 25.36 | 24.60 | 28.73 | 29.46 | 27.63 | 21.56 | 26.94 | 22.95 | 24.55 | 27.07 | 26.75 | 26.71 |
|  | Lateral root_1 | 21.40 | 24.54 | 21.45 | 21.27 | 25.39 | 23.58 | 23.40 | 19.63 | 21.75 | 20.85 | 21.22 | 25.89 | 22.91 | 23.52 |
|  | Lateral root_2 | 26.45 | 28.98 | 26.60 | 25.48 | 29.55 | 31.03 | 28.43 | 21.80 | 27.67 | 24.08 | 25.61 | 25.11 | 27.94 | 27.58 |
|  | Lateral root_3 | 29.89 | 30.87 | 27.78 | 28.44 | 31.22 | 35.00 | 31.95 | 24.73 | 33.05 | 26.31 | 28.64 | 28.89 | 30.66 | 30.39 |
|  | Leaf_1 | 19.48 | 23.52 | 19.19 | 20.15 | 23.01 | 21.66 | 22.60 | 18.64 | 21.57 | 20.20 | 20.61 | 28.52 | 21.92 | 22.84 |
|  | Leaf_2 | 19.78 | 23.60 | 19.01 | 20.48 | 22.85 | 21.71 | 22.69 | 18.77 | 21.50 | 20.71 | 20.94 | 22.85 | 22.08 | 22.97 |
|  | Leaf_3 | 19.91 | 23.59 | 19.19 | 20.28 | 22.82 | 21.68 | 22.86 | 18.68 | 21.60 | 20.47 | 20.48 | 22.87 | 22.03 | 22.75 |
| Rapid growth and flowering stage (May) | Taproot_1 | 22.65 | 24.94 | 22.07 | 21.98 | 25.69 | 24.21 | 25.11 | 18.91 | 24.29 | 20.77 | 21.88 | 23.65 | 23.56 | 23.69 |
|  | Taproot_2 | 24.42 | 26.61 | 23.81 | 23.51 | 27.16 | 27.12 | 26.53 | 19.63 | 25.63 | 21.85 | 23.74 | 24.60 | 25.47 | 25.47 |
|  | Taproot_3 | 26.11 | 27.34 | 24.68 | 24.02 | 28.18 | 27.77 | 27.68 | 20.60 | 26.64 | 22.20 | 24.39 | 25.70 | 26.24 | 26.20 |
|  | Lateral root_1 | 27.92 | 29.56 | 25.91 | 26.95 | 30.13 | 31.19 | 29.71 | 24.66 | 28.59 | 26.49 | 27.39 | 27.11 | 28.60 | 28.86 |
|  | Lateral root_2 | 23.97 | 26.18 | 22.83 | 23.09 | 26.77 | 27.22 | 26.05 | 20.71 | 24.57 | 22.56 | 23.23 | 27.94 | 24.57 | 24.99 |
|  | Lateral root_3 | 25.52 | 26.68 | 22.91 | 23.99 | 27.58 | 27.05 | 26.78 | 22.50 | 24.94 | 23.66 | 24.04 | 25.73 | 25.44 | 25.76 |
|  | Leaf_1 | 24.30 | 26.77 | 22.70 | 23.87 | 27.23 | 27.54 | 28.34 | 22.99 | 26.06 | 23.86 | 23.53 | 26.50 | 24.47 | 25.30 |
|  | Leaf_2 | 19.84 | 23.16 | 20.50 | 20.22 | 23.55 | 23.95 | 24.14 | 19.13 | 22.72 | 20.26 | 19.55 | 25.20 | 21.09 | 22.12 |
|  | Leaf_3 | 19.71 | 22.75 | 19.62 | 19.79 | 22.70 | 23.35 | 23.09 | 18.50 | 22.21 | 19.63 | 19.22 | 22.49 | 20.98 | 21.97 |
| Seed setting stage (July) | Taproot_1 | 21.19 | 25.29 | 22.46 | 21.11 | 25.66 | 26.42 | 23.88 | 17.65 | 23.08 | 20.44 | 21.99 | 22.79 | 24.02 | 23.92 |
|  | Taproot_2 | 21.32 | 25.65 | 22.82 | 21.40 | 25.73 | 26.76 | 23.98 | 17.11 | 24.23 | 20.77 | 22.38 | 24.66 | 24.37 | 23.90 |
|  | Taproot_3 | 22.85 | 27.09 | 24.55 | 22.92 | 27.27 | 29.18 | 25.61 | 18.76 | 25.72 | 21.46 | 23.91 | 25.49 | 25.90 | 25.27 |
|  | Lateral root_1 | 24.71 | 27.58 | 23.96 | 23.97 | 27.91 | 29.12 | 26.97 | 20.29 | 27.60 | 22.63 | 25.08 | 26.76 | 26.34 | 26.46 |
|  | Lateral root_2 | 31.03 | 31.82 | 29.21 | 29.02 | 33.83 | 35.00 | 32.46 | 25.50 | 31.87 | 27.87 | 29.85 | 28.87 | 30.94 | 30.77 |
|  | Lateral root_3 | 27.87 | 29.70 | 26.59 | 26.50 | 30.87 | 32.06 | 29.71 | 22.90 | 29.74 | 25.25 | 27.47 | 27.81 | 28.64 | 28.62 |
|  | Leaf_1 | 21.29 | 23.75 | 20.08 | 20.64 | 24.06 | 25.60 | 25.88 | 18.60 | 24.25 | 19.90 | 20.51 | 28.96 | 22.28 | 22.80 |
|  | Leaf_2 | 21.45 | 24.04 | 21.04 | 20.77 | 24.23 | 25.43 | 26.05 | 18.91 | 25.13 | 20.59 | 20.10 | 23.75 | 21.97 | 22.87 |
|  | Leaf_3 | 23.35 | 25.14 | 21.52 | 22.31 | 25.20 | 26.23 | 26.72 | 19.50 | 25.41 | 21.41 | 21.74 | 23.96 | 23.13 | 23.82 |
| Senescence stage (October) | Taproot_1 | 23.87 | 25.88 | 22.95 | 22.09 | 27.05 | 27.80 | 25.85 | 18.94 | 25.76 | 20.43 | 23.49 | 24.85 | 25.87 | 25.45 |
|  | Taproot_2 | 25.40 | 27.63 | 24.96 | 24.55 | 29.18 | 29.75 | 27.81 | 20.60 | 27.38 | 22.18 | 25.19 | 25.66 | 27.61 | 27.49 |
|  | Taproot_3 | 21.67 | 24.42 | 20.90 | 20.66 | 25.27 | 26.00 | 24.05 | 18.00 | 23.81 | 19.15 | 22.01 | 25.81 | 24.08 | 23.94 |
|  | Lateral root_1 | 23.05 | 25.91 | 22.36 | 21.79 | 26.57 | 27.37 | 25.86 | 19.68 | 25.25 | 21.12 | 23.14 | 24.18 | 25.35 | 25.02 |
|  | Lateral root_2 | 27.23 | 27.94 | 23.87 | 24.82 | 29.13 | 31.55 | 28.71 | 22.71 | 30.54 | 24.17 | 26.58 | 26.03 | 28.21 | 28.17 |
|  | Lateral root_3 | 23.68 | 26.02 | 23.17 | 22.64 | 27.15 | 28.25 | 25.78 | 19.28 | 25.65 | 21.42 | 23.33 | 27.16 | 25.92 | 25.46 |

**Table S2. Cq values under different experimental conditions in *G. inflata*.**

|  | **Conditions** | ***ACT*** | ***CAC*** | ***CYP*** | ***DNAJ*** | ***DREB*** | ***EF1*** | ***RAN*** | ***TIF1*** | ***TUB*** | ***UBC2*** | ***ABCC2*** | ***COPS3*** | ***CS*** | ***R3HDM2*** |
| --- | --- | --- | --- | --- | --- | --- | --- | --- | --- | --- | --- | --- | --- | --- | --- |
| Returning green stage (April) | Taproot_1 | 19.92 | 25.23 | 25.58 | 22.22 | 25.91 | 24.45 | 25.27 | 18.18 | 26.75 | 20.18 | 22.93 | 23.10 | 24.99 | 24.53 |
|  | Taproot_2 | 21.89 | 25.80 | 25.06 | 22.71 | 26.14 | 26.26 | 25.82 | 19.13 | 26.92 | 20.17 | 21.91 | 24.98 | 25.47 | 25.08 |
|  | Taproot_3 | 20.12 | 24.81 | 25.55 | 23.51 | 25.80 | 25.88 | 24.59 | 19.35 | 26.79 | 20.47 | 22.03 | 25.02 | 24.34 | 25.36 |
|  | Lateral root_1 | 19.21 | 25.16 | 25.75 | 24.57 | 25.15 | 27.04 | 27.98 | 22.12 | 26.67 | 19.86 | 22.09 | 23.52 | 24.85 | 24.55 |
|  | Lateral root_2 | 19.61 | 24.25 | 24.67 | 22.05 | 24.63 | 27.95 | 24.79 | 19.98 | 24.42 | 19.28 | 20.08 | 25.51 | 24.38 | 25.77 |
|  | Lateral root_3 | 18.23 | 23.64 | 24.21 | 21.50 | 24.64 | 25.62 | 26.13 | 19.11 | 23.26 | 20.18 | 22.13 | 23.19 | 23.69 | 24.69 |
|  | Leaf_1 | 17.55 | 23.88 | 20.79 | 21.04 | 24.73 | 22.82 | 24.73 | 21.04 | 22.07 | 21.02 | 21.38 | 22.68 | 22.31 | 23.13 |
|  | Leaf_2 | 18.10 | 22.98 | 21.66 | 20.11 | 23.89 | 20.50 | 22.10 | 17.71 | 20.61 | 20.91 | 22.36 | 20.52 | 23.03 | 21.78 |
|  | Leaf_3 | 18.31 | 24.86 | 20.35 | 19.91 | 24.07 | 21.33 | 21.49 | 20.52 | 20.95 | 21.08 | 21.63 | 21.05 | 22.35 | 22.27 |
| Rapid growth and flowering stage (May) | Taproot_1 | 21.72 | 25.29 | 22.90 | 22.07 | 25.23 | 24.54 | 24.03 | 19.30 | 22.93 | 20.59 | 22.09 | 23.72 | 23.85 | 23.87 |
|  | Taproot_2 | 22.69 | 25.76 | 23.55 | 23.14 | 25.52 | 23.84 | 24.88 | 18.90 | 24.94 | 20.93 | 22.36 | 22.69 | 24.62 | 22.85 |
|  | Taproot_3 | 22.61 | 25.59 | 23.10 | 22.90 | 26.48 | 24.16 | 24.44 | 20.03 | 22.73 | 21.59 | 21.77 | 23.15 | 23.79 | 23.26 |
|  | Lateral root_1 | 24.43 | 27.12 | 23.74 | 24.49 | 27.68 | 27.07 | 26.04 | 21.17 | 24.59 | 23.48 | 23.40 | 24.06 | 25.21 | 24.51 |
|  | Lateral root_2 | 21.37 | 26.23 | 22.99 | 23.51 | 26.87 | 27.01 | 24.42 | 20.08 | 24.22 | 21.38 | 23.06 | 24.43 | 24.97 | 24.82 |
|  | Lateral root_3 | 23.67 | 25.20 | 21.68 | 22.84 | 24.66 | 26.22 | 24.17 | 22.14 | 22.56 | 21.57 | 22.87 | 22.73 | 24.54 | 23.33 |
|  | Leaf_1 | 20.14 | 26.08 | 21.68 | 23.61 | 25.07 | 25.73 | 25.87 | 22.64 | 25.06 | 21.88 | 22.09 | 23.53 | 23.13 | 23.16 |
|  | Leaf_2 | 22.54 | 26.44 | 21.99 | 23.94 | 24.27 | 25.73 | 25.74 | 24.19 | 24.52 | 21.35 | 21.00 | 23.26 | 22.30 | 22.50 |
|  | Leaf_3 | 21.13 | 26.53 | 22.08 | 23.66 | 24.77 | 24.30 | 25.65 | 22.86 | 24.78 | 21.39 | 21.66 | 22.50 | 22.90 | 22.97 |
| Seed setting stage (July) | Taproot_1 | 23.14 | 25.63 | 23.99 | 22.20 | 26.09 | 24.94 | 25.00 | 19.28 | 25.59 | 21.34 | 23.37 | 23.56 | 24.71 | 23.58 |
|  | Taproot_2 | 22.07 | 24.01 | 22.83 | 20.73 | 24.94 | 23.47 | 23.32 | 19.17 | 22.56 | 20.02 | 21.86 | 22.07 | 23.25 | 22.46 |
|  | Taproot_3 | 22.69 | 24.35 | 23.36 | 20.69 | 25.19 | 24.40 | 23.32 | 18.11 | 22.76 | 20.53 | 22.18 | 23.09 | 23.96 | 23.12 |
|  | Lateral root_1 | 21.57 | 25.20 | 19.96 | 21.77 | 26.05 | 25.58 | 24.77 | 20.69 | 24.61 | 21.42 | 23.38 | 23.55 | 24.94 | 25.04 |
|  | Lateral root_2 | 25.15 | 26.59 | 24.64 | 23.39 | 27.06 | 27.41 | 26.25 | 21.47 | 24.94 | 22.84 | 24.75 | 25.13 | 26.74 | 25.73 |
|  | Lateral root_3 | 26.38 | 30.45 | 26.01 | 26.34 | 28.92 | 29.43 | 29.24 | 21.24 | 30.93 | 24.42 | 27.02 | 26.85 | 28.27 | 27.15 |
|  | Leaf_1 | 22.45 | 27.68 | 20.38 | 24.05 | 25.30 | 24.70 | 27.36 | 18.28 | 25.09 | 21.34 | 22.10 | 23.52 | 23.30 | 22.30 |
|  | Leaf_2 | 23.02 | 24.99 | 20.28 | 22.22 | 25.17 | 24.43 | 25.36 | 19.28 | 24.72 | 21.11 | 22.43 | 23.49 | 23.30 | 23.73 |
|  | Leaf_3 | 22.13 | 24.59 | 20.14 | 21.56 | 24.48 | 23.68 | 25.12 | 20.08 | 24.84 | 20.54 | 21.41 | 22.97 | 22.59 | 23.17 |
| Senescence stage (October) | Taproot_1 | 22.36 | 25.54 | 23.71 | 21.71 | 26.21 | 26.16 | 25.56 | 18.91 | 26.27 | 19.60 | 22.96 | 23.88 | 24.61 | 24.08 |
|  | Taproot_2 | 21.62 | 24.63 | 22.28 | 21.04 | 26.20 | 25.54 | 24.53 | 18.05 | 24.82 | 19.47 | 22.15 | 23.43 | 23.89 | 23.61 |
|  | Taproot_3 | 21.95 | 24.82 | 22.83 | 21.56 | 25.30 | 25.10 | 24.71 | 17.75 | 25.38 | 18.80 | 21.91 | 23.45 | 23.87 | 23.71 |
|  | Lateral root_1 | 21.91 | 25.61 | 22.86 | 22.04 | 25.81 | 26.81 | 25.26 | 18.89 | 25.39 | 19.76 | 22.40 | 23.05 | 24.08 | 23.60 |
|  | Lateral root_2 | 21.92 | 25.13 | 22.11 | 22.71 | 25.32 | 23.84 | 25.58 | 18.34 | 25.86 | 19.93 | 21.98 | 23.27 | 23.26 | 23.33 |
|  | Lateral root_3 | 20.93 | 24.74 | 21.98 | 21.25 | 25.88 | 25.56 | 24.49 | 20.09 | 24.58 | 19.95 | 22.25 | 23.59 | 23.93 | 18.28 |

**Table S3. Cq values of candidate reference genes under different conditions in *G. uralensis*.**

| **Gene symbol** | **Total** | **Different developmental stages** | | | | **Different tissues** | | |
| --- | --- | --- | --- | --- | --- | --- | --- | --- |
|  |  | **Returning green stage (April)** | **Rapid growth and flowering stage (May)** | **Seed setting stage (July)** | **Aging stage (October)** | **Roots** | **Rhizomes** | **Leaves** |
| *ACT* | 23.77±0.52 | 23.34±1.21 | 23.83±0.91 | 23.89±1.15 | 24.15±0.80 | 23.55±0.51 | 26.06±0.83 | 21.01±0.81 |
| *CAC* | 26.31±0.39 | 26.27±0.91 | 26.00±0.70 | 26.67±0.89 | 26.30±0.60 | 26.34±0.35 | 27.98±0.64 | 24.04±0.55 |
| *CYP* | 23.11±0.44 | 23.03±1.15 | 22.78±0.65 | 23.58±0.96 | 23.04±0.64 | 23.60±0.41 | 24.72±0.70 | 20.32±0.56 |
| *DNAJ* | 23.03±0.42 | 23.05±0.96 | 23.05±0.72 | 23.18±0.96 | 22.76±0.64 | 22.80±0.42 | 24.83±0.73 | 20.94±0.54 |
| *DREB* | 26.86±0.46 | 26.47±1.06 | 26.55±0.76 | 27.20±1.09 | 27.39±0.78 | 27.05±0.39 | 28.84±0.69 | 23.96±0.72 |
| *EF1* | 27.50±0.59 | 26.85±1.64 | 26.60±0.81 | 28.42±1.09 | 28.45±0.84 | 27.67±0.50 | 29.87±0.98 | 24.13±1.02 |
| *RAN* | 26.35±0.43 | 25.88±1.10 | 26.38±0.69 | 26.81±0.91 | 26.34±0.54 | 25.96±0.44 | 27.98±0.77 | 24.71±0.71 |
| *TIF1* | 20.30±0.37 | 20.43±0.71 | 20.85±0.70 | 19.91±0.89 | 19.87±0.56 | 19.32±0.43 | 22.03±0.62 | 19.30±0.52 |
| *TUB* | 25.69±0.50 | 25.18±1.32 | 25.07±0.65 | 26.34±0.96 | 26.40±0.82 | 25.50±0.40 | 27.60±0.96 | 23.38±0.88 |
| *UBC2* | 22.09±0.37 | 22.12±0.71 | 22.36±0.70 | 22.26±0.88 | 21.41±0.59 | 21.31±0.35 | 23.87±0.66 | 20.78±0.48 |
| *ABCC2* | 23.41±0.45 | 23.20±0.95 | 23.00±0.84 | 23.67±1.09 | 23.96±0.85 | 23.36±0.36 | 25.47±0.75 | 20.74±0.71 |
| *COPS3* | 25.70±0.32 | 25.84±0.71 | 25.44±0.56 | 25.89±0.77 | 25.62±0.69 | 25.13±0.32 | 26.79±0.42 | 25.01±0.74 |
| *CS* | 25.19±0.46 | 25.15±1.05 | 24.49±0.80 | 25.29±1.00 | 26.17±0.91 | 25.50±0.38 | 27.13±0.71 | 22.22±0.74 |
| *R3HDM2* | 25.35±0.40 | 25.35±0.88 | 24.93±0.71 | 25.38±0.92 | 25.92±0.77 | 25.29±0.37 | 27.13±0.66 | 23.05±0.61 |

Cq values are presented as Cq ± SE.

**Table S4. Cq values of candidate reference genes under different conditions in *G. inflata***.

| **Gene symbol** | **Total** | **Different developmental stages** | | | | **Different tissues** | | |
| --- | --- | --- | --- | --- | --- | --- | --- | --- |
|  |  | **Returning green stage (April)** | **Rapid growth and flowering stage (May)** | **Seed setting stage (July)** | **Aging stage (October)** | **Roots** | **Rhizomes** | **Leaves** |
| *ACT* | 21.59±0.35 | 19.22±0.45 | 22.25±0.44 | 23.18±0.52 | 21.78±0.24 | 21.90±0.29 | 22.03±0.71 | 20.60±0.63 |
| *CAC* | 25.42±0.23 | 24.51±0.30 | 26.03±0.21 | 25.94±0.68 | 25.08±0.39 | 25.12±0.17 | 25.78±0.51 | 25.34±0.42 |
| *CYP* | 22.87±0.30 | 23.73±0.73 | 22.63±0.26 | 22.40±0.76 | 22.63±0.53 | 23.73±0.32 | 23.38±0.52 | 21.04±0.31 |
| *DNAJ* | 22.46±0.24 | 21.96±0.51 | 23.35±0.24 | 22.55±0.60 | 21.72±0.37 | 22.04±0.27 | 23.04±0.43 | 22.23±0.48 |
| *DREB* | 25.56±0.18 | 25.00±0.27 | 25.62±0.38 | 25.91±0.45 | 25.79±0.23 | 25.75±0.14 | 26.06±0.39 | 24.64±0.21 |
| *EF1* | 25.20±0.31 | 24.65±0.86 | 25.40±0.41 | 25.34±0.64 | 25.50±0.42 | 24.89±0.26 | 26.63±0.41 | 23.69±0.62 |
| *RAN* | 25.09±0.26 | 24.77±0.66 | 25.03±0.27 | 25.53±0.63 | 25.02±0.35 | 24.62±0.23 | 25.76±0.44 | 24.82±0.54 |
| *TIF1* | 19.94±0.28 | 19.68±0.46 | 21.26±0.60 | 19.73±0.40 | 18.67±0.34 | 18.85±0.20 | 20.44±0.36 | 20.73±0.68 |
| *TUB* | 24.64±0.34 | 24.27±0.88 | 24.04±0.34 | 25.12±0.81 | 25.38±0.23 | 24.87±0.49 | 25.17±0.61 | 23.63±0.59 |
| *UBC2* | 20.86±0.20 | 20.35±0.20 | 21.57±0.27 | 21.51±0.45 | 19.59±0.33 | 20.31±0.23 | 21.17±0.48 | 21.18±0.22 |
| *ABCC2* | 22.39±0.20 | 21.84±0.26 | 22.25±0.25 | 23.17±0.59 | 22.28±0.17 | 22.29±0.15 | 22.95±0.49 | 21.78±0.16 |
| *COPS3* | 23.47±0.21 | 23.29±0.58 | 23.34±0.22 | 23.80±0.46 | 23.45±0.11 | 23.51±0.24 | 24.07±0.35 | 22.61±0.33 |
| *CS* | 24.10±0.22 | 23.94±0.39 | 23.92±0.33 | 24.56±0.62 | 23.94±0.24 | 24.28±0.18 | 24.90±0.40 | 22.80±0.20 |
| *R3HDM2* | 23.68±0.27 | 24.13±0.47 | 23.47±0.26 | 24.03±0.54 | 22.77±0.73 | 23.79±0.25 | 24.23±0.63 | 22.78±0.49 |

Cq values are presented as Cq ± SE.

**Table S5. Recommended reference genes for RT-qPCR analysis under different conditions among Leguminosae plants.**

| **Species** | **Candidates** | **Recommendation** | **Software/algorism** | **Reference** |
| --- | --- | --- | --- | --- |
| ***Tissues × Developmental stages*** | | | | |
| *Glycyrrhiza uralensis* F. | *ACT, CAC, CYP, DNAJ, DREB, EF1, RAN, TIF1, TUB, UBC2, ABCC2, COPS3, CS, R3HDM2* | *CAC*, *R3HDM2*, *DNAJ* | geNorm, NormFinder, BestKeeper, ∆Ct, RefFinder | This study |
| *Glycyrrhiza inflata* B. | *ACT, CAC, CYP, DNAJ, DREB, EF1, RAN, TIF1, TUB, UBC2, ABCC2, COPS3, CS, R3HDM2* | *COPS3*, *DREB*, *ABCC2* | geNorm, NormFinder, BestKeeper, ∆Ct, RefFinder | This study |
| *Glycine max* (L.) Merr. | *ELF1A, ELF1B, TUB, CYP2, ACT11, TUA5, G6PD, UBC2, UBC4* | *UBC4, UBC2, CYP* | geNorm, NormFinder | 37 |
| ***Developmental stages*** | | | | |
| *Glycyrrhiza uralensis* F. | *ACT, CAC, CYP, DNAJ, DREB, EF1, RAN, TIF1, TUB, UBC2, ABCC2, COPS3, CS, R3HDM2* | *CAC*, *CYP*, *ABCC2* | geNorm, NormFinder, BestKeeper, ∆Ct, RefFinder | This study |
| *Glycyrrhiza inflata* B. | *ACT, CAC, CYP, DNAJ, DREB, EF1, RAN, TIF1, TUB, UBC2, ABCC2, COPS3, CS, R3HDM2* | *RAN*, *COPS3*, *CS* | geNorm, NormFinder, BestKeeper, ∆Ct, RefFinder | This study |
| *Arachis hypogaea* L. | *G6PD, ADH3, LEC, ACT11, ATPsyn, CYP2, ELF1B, LEC, UBC1* | *ADH3, G6PD, ELF1B* | geNorm, NormFinder | 47 |
| *Cyamopsis tetragonoloba* L.Taub | *ACT11, UBC2, ACT7, TUA, CYP, EF-1α, TUB, UBQ10, GAPDH, 18S rRNA* | *ACT11, UBC2, ACT7* | geNorm, NormFinder, BestKeeper, ∆Ct, RefFinder | 40 |
| *Eremosparton songoricum* | *EF, ACT, 18S, GAPDH, UBQ, β-TUB1, β-TUB2, α-TUB, DREB* | *EF, ACT, α-TUB* | geNorm | 36 |
| *Glycine max* (L.) Merr. | *SKIP16, UKN1, MTP, CYP, UKN2, ACT2/7, ACT11, TUB4, TUA5, UBQ10, EF1b, PEPKR1, HDC, TIP41* | *SKIP16, UKN1, MTP* | geNorm, NormFinder | 42 |
| *Glycine max* (L.) Merr. | *ELF1A, ELF1B, TUB, CYP2, ACT11, TUA5, G6PD, UBC2, UBC4* | *ELF1A, ELF1B, TUA5* | geNorm, NormFinder | 37 |
| *Hedysarum coronarium* L. | *TUA1, TUA2, and UBQ, 18S rRNA, ΒACT, Helicase, GH720838* | *TUA1, TUA2, UBQ* | geNorm, BestKeeper | 39 |
| *Lens culinaris* Medic. | *RPL2, Tub, Rbcl, HSP70, Mat K, 18S rRNA, GAPDH, EF1a* | *HSP70, RPL2, Rbcl* | geNorm, BestKeeper, NormFinder, RefFinder | 48 |
| *Lupinus angustifolius* L. | *UBC , HEL, PTB, TUB, PDF2, PPR, PUL7* | *UBC, HEL, PTB* | geNorm, NormFinder, BestKeeper, ∆Ct, RefFinder | 41 |
| *Cicer arietinum* L. | *ACT1, EF1a, GAPDH, IF4a, TUB6, UBC, UBQ5, UBQ10, 18SrRNA, 25SrRNA, GRX, HSP90* | *EF1a, HSP90, IF4a* | geNorm | 49 |
| ***Tissues*** | | | | |
| *Glycyrrhiza uralensis* F. | *ACT, CAC, CYP, DNAJ, DREB, EF1, RAN, TIF1, TUB, UBC2, ABCC2, COPS3, CS, R3HDM2* | *R3HDM2*, *CAC*, *TUB* | geNorm, NormFinder, BestKeeper, ∆Ct, RefFinder | This study |
| *Glycyrrhiza inflata* B. | *ACT, CAC, CYP, DNAJ, DREB, EF1, RAN, TIF1, TUB, UBC2, ABCC2, COPS3, CS, R3HDM2* | *COPS3*, *R3HDM2*, *DREB* | geNorm, NormFinder, BestKeeper, ∆Ct, RefFinder | This study |
| *Arachis hypogaea* L. | *ACT11,TUA5, UKN2, PEPKR1, TIP41, TUB4, UKN1, ACT2/7, EF1b, CYP, UBQ10, HDC, SKIP16, MTP* | *UKN2, TUA5, ACT11* | geNorm, NormFinder | 47 |
| *Arachis hypogaea* L. | *UBI1, GAPDH, ELF1B, G6PD, ACTIN11, ACTIN7, ACTIN1, ADH3, UKN2, YLS8, 60S* | *UBI1, GAPDH, ELF1B* | geNorm, NormFinder, BestKeeper | 50 |
| *Cassia obtusifolia* L. | *EF1α2, UBQ1, UBQ2, α-TUB2, CYP1, ACT1, ACT2, CYP2, α-TUB1, β-TUB2* | *EF1α2, UBQ1, CYP1* | geNorm, NormFinder, BestKeeper | 44 |
| *Cicer arietinum* L. | *PP2A, ABCT, PPR, G6PD, PEX4, YLS8, ELF1β, hsp80, CAC, GAPDH, IF3, TIP41, UCP, ASMDM, CDPK, VPS, UPL7, ADH3, FBOX, SAND, IF4α, UNK, ELF1α, SKIP16, CYP* | *PP2A, ABCT, VPS* | geNorm, NormFinder | 38 |
| *Cyamopsis tetragonoloba* L.Taub | *CYP , ACT11, TUB, EF-1α, TUA, ACT 7, UBQ10, UBC 2, GAPDH, 18S rRNA* | *CYP , ACT11, 18S Rrna* | geNorm, NormFinder, BestKeeper, ∆Ct, RefFinder | 40 |
| *Eremosparton songoricum* | *EF, ACT, 18S, GAPDH, UBQ, β-TUB1, β-TUB2, α-TUB, DREB* | *ACT, β-TUB2, UBQ* | geNorm | 36 |
| *Glycine max* (L.) Merr. | *ACT11, UKN1, UKN2, ACT2/7, SKIP16, TUB4, TUA5, CYP, UBQ10, EF1b, MTP, PEPKR1, HDC, TIP41* | *ACT11, UKN1, UKN2* | geNorm, NormFinder | 42 |
| *Glycine max* (L.) Merr. | *CYP2, UBC4, TUB, ELF1A, ELF1B, ACT11, TUA5, G6PD, UBC2* | *ELF1A, UBC2, ACT11* | geNorm, NormFinder | 37 |
| *Glycine max* (L.) Merr. | *CYP2, UBC4, TUB, ELF1A, ELF1B, ACT11, TUA5, G6PD, UBC2* | *ACT11, TUA5, ELF1A* | geNorm, NormFinder | 37 |
| *Glycine max* (L.) Merr. | *CYP2, UBC4, TUB, ELF1A, ELF1B, ACT11, TUA5, G6PD, UBC2* | *CYP2, ELF1A, ACT11* | geNorm, NormFinder | 37 |
| *Lupinus angustifolius* L. | *UBC , HEL, PTB, TUB, PDF2, PPR, PUL7* | *UBC, HEL, PTB* | geNorm, NormFinder, BestKeeper, ∆Ct, RefFinder | 41 |
| *Medicago sativa* L. | *Msc27, 18S rRNA, GAPDH, ELF-1α, β -Actin, E2 ubiquitin-conjugating enzyme, TUB, Actin2* | *Msc27, 18S rRNA, EF-1α* | geNorm, BestKeeper | 45 |
| *Phaseolus vulgaris* Linn. | *β-tubulin, T197, Act, 18S, Cons 6, Cons 7, Cons 15, UBC9, Tc127, Tc185* | *β-tubulin, T197, Act* | geNorm, NormFinder, BestKeeper, ∆Ct, RefFinder | 43 |
| *Vigna angularis* (Willd.) Ohwi et Ohashi | *EF, UBN, ACT, Fbox, ZMPP, GAPDH, PP2A, UBC, PTB* | *UBN, EF, Fbox* | geNorm, NormFinder, BestKeeper | 51 |
